# Maxillary Osteomyelitis in a Patient with Pansinusitis and Recently Diagnosed Focal Segmental Glomerulosclerosis

**DOI:** 10.7759/cureus.5347

**Published:** 2019-08-08

**Authors:** Gagandeep S Gill, Maren Pulcini

**Affiliations:** 1 Medicine, Nova Southeastern University College of Osteopathic Medicine, Fort Lauderdale, USA; 2 Family Medicine, Henry Ford Hospital System, Detroit, USA

**Keywords:** omadacycline, focal segmental glomerulosclerosis, pansinusitis, osteomyelitis, african american, infectious diseases, sinusitis, maxillary osteomyelitis, fsgs, medicine

## Abstract

Given the rarity of osteomyelitis of the maxilla, a confirmed diagnosis requires extensive investigation into the possible underlying causes of the disease. The most common causes of osteomyelitis of the jaw are periodontal infections and sinusitis with contiguous seeding and infection of the bone. Even in those affected by these potential causes, an immunocompromised state is usually present for an extensive infection to occur. Early recognition is key for ensuring appropriate treatment and avoidance of life-threatening complications. We report a case of maxillary osteomyelitis in a patient with no clear predisposing risk factors, a history of recurrent pansinusitis, and recently diagnosed focal segmental glomerulosclerosis. Cultures of the bone revealed multiple bacterial and fungal organisms. He was treated with surgical debridement, teeth extraction, and a prolonged course of antifungals and antibiotics.

## Introduction

Osteomyelitis is defined as an infection of the bone and usually occurs secondary to either hematogenous or non-hematogenous seeding of the involved bone by bacteria. In the case of osteomyelitis of the jaw, it usually results from odontogenic infections that spread contiguously and infect bone [1]. Although osteomyelitis of the jaw is a relatively uncommon phenomenon, the mandible is much more susceptible to infection as compared to the maxilla due to the fact that the cortical plates of the mandible are thin and blood supply to the tissue in the medullary area is poor [2]. When osteomyelitis of the jawbones does occur, there is usually an identifiable predisposing factor, such as fracture, history of irradiation, diabetes mellitus, human immunodeficiency virus (HIV), steroid therapy, malnutrition, chemotherapy, or other causes, leading to an immunocompromised status [2]. The most common presenting symptom of maxillary osteomyelitis is severe mandibular pain, which may be accompanied by anesthesia or hypoesthesia of the affected side [3]. We hereby report a case of maxillary osteomyelitis in an African American male with recurrent pansinusitis and recently diagnosed focal segmental glomerulosclerosis (FSGS) with no clear predisposing risk factors.

## Case presentation

A 45-year-old male presented to the emergency department (ED) with a complaint of bilateral epistaxis. His past medical history was significant for hypertension, recurrent sinusitis, and recently diagnosed FSGS. Of note, he had recently been treated for a dental abscess with both broad-spectrum intravenous (IV) antibiotics followed by a 14-day course of oral Augmentin. History revealed that he had not had regular follow-up with a dentist as advised. He was found to be febrile and tachycardic on presentation and there was a concern for sepsis. On examination, the patient endorsed right-sided jaw pain but denied discharge, nasal congestion, sinus pressure, or sinus pain. His neurological evaluation was within normal limits, and cervical lymphadenopathy was absent. The oropharyngeal examination was notable for mild purulent discharge from the first through third molars associated with tenderness, along with erosions and erythema of the hard palate. He reported no previous history of drug use.

**Figure 1:**
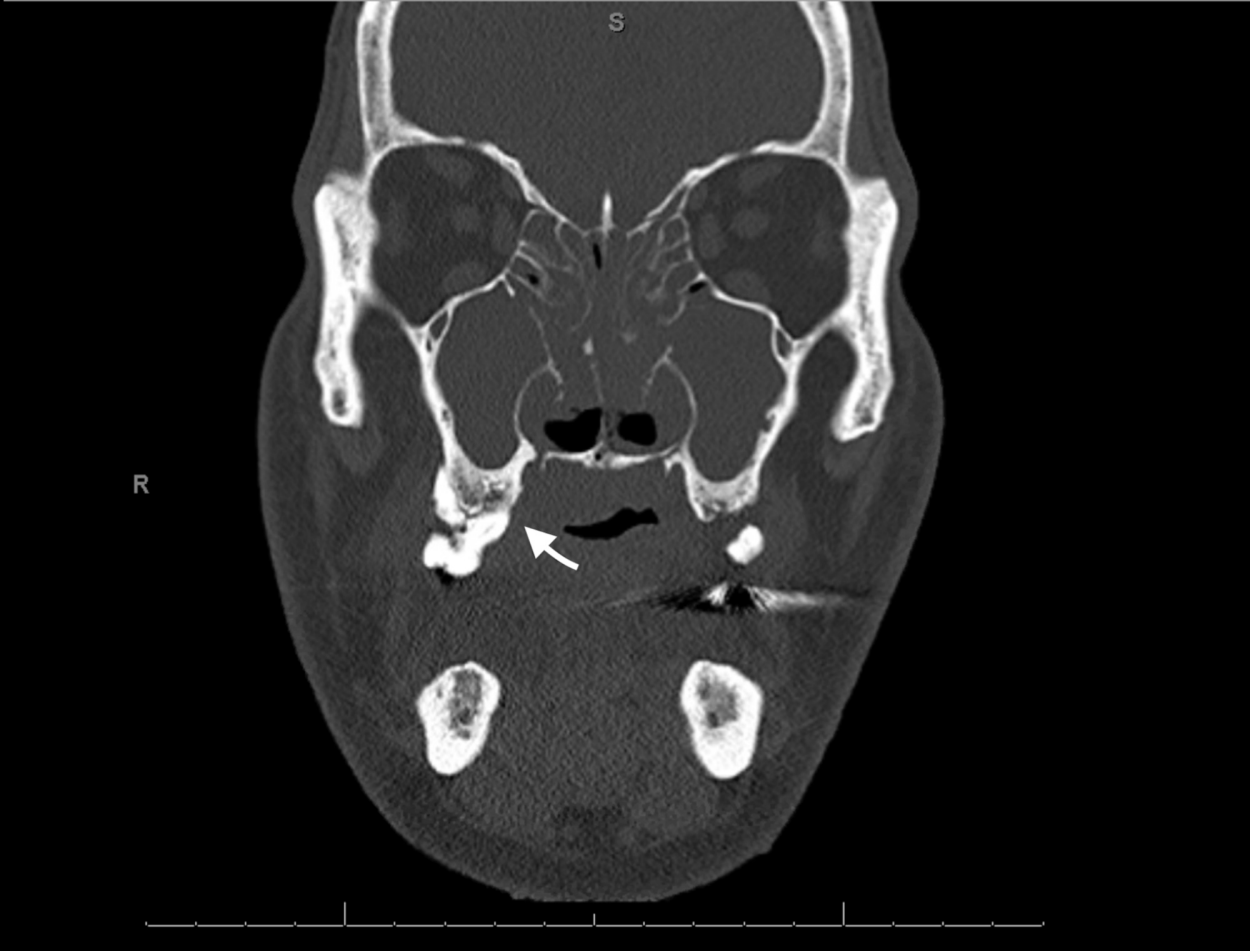
CT maxillofacial w/o contrast on presentation

Computed tomography (CT) maxillofacial (Figure 1) was performed, which revealed completely opacified maxillary sinuses, opacification of the paranasal sinuses, and numerous gas bubbles present within the sphenoid. Additionally, there was a large nasal septal perforation with debris present within the nasal cavity as well as within the pharynx, with numerous gas bubbles. In addition, there was mucoperiosteal thickening involving the maxillary sinuses, pterygoids, and sphenoids, with extensive periodontal disease and loss of the right maxillary bicuspid. Lastly, there was an extensive abnormality in the maxillary bone (Figure 1) extending through the hard palate, concerning for osteomyelitis. He subsequently was put on intravenous (IV) antibiotics and underwent a surgical evaluation that revealed a necrotic right maxillary bone. The surgical intervention included extensive debridement of the necrotic tissue in addition to the extraction of several teeth (#4, 5, and 7); biopsies of the bone and palate were taken. Bacterial culture of the biopsied specimens revealed alpha-hemolytic strep, E. coli, candida albicans, and group b Streptococci species. Over the course of his hospital stay his human immunodeficiency virus (HIV), hepatitis C, and autoimmune workup (ANA, c-ANCA, and p-ANCA) were negative. C3 was within normal limits and C4 was slightly elevated at 57 mg/dL. While in hospital, in addition to bone debridement, the patient was treated with IV antibiotics and antifungals and was discharged with oral medication for a total of six weeks of therapy. Of note, he had not received any treatment (including steroids) for his FSGS prior to presentation.

## Discussion

In today’s era of advanced diagnostics and antibiotics, osteomyelitis of the maxilla remains a very rare occurrence, especially in patients who are immunocompetent and free of any apparent predisposing risk factors. When pansinusitis is suspected to be the cause of facial cellulitis, it is usually the frontal bones that are affected and very rarely the maxilla [4]. In the event that maxillary osteomyelitis does occur, dental infection and sinusitis are the two most common causes but are still often associated with some kind of immunocompromised state such as diabetes [5]. In fact, some studies have found that up to 68% of cases, maxillary sinusitis is associated with hyperglycemia secondary to uncontrolled diabetes mellitus [5]. In this case, however, although the patient had both a history of dental disease and pansinusitis, he did not have any previously known risk factors, including diabetes mellitus, malignancy, irradiation, chemotherapy, autoimmune disease, steroid use, hepatitis, or HIV. Antinuclear antibody screen for possible systemic lupus erythematous, sometimes associated with FSGS and immunosuppression, was also negative [6]. Sadallah et al. (1999) have previously reported a possible association between complement factor I deficiency in patients with glomerulonephritis. This deficiency can lead to uncontrolled activation of the alternative complement pathway, ultimately leading to the depletion of several pathway components, more specifically C3 [7]. Depletion of C3 and related components in this pathway is associated with an increased predisposition to pyogenic infections [7]. In this case, deficiencies in the complement pathway were also explored as a possible predisposing factor to the patient’s maxillary osteomyelitis and recurrent sinusitis. C3 levels were found to be within normal limits and C4 was found to be elevated.

Successful treatment of osteomyelitis can be achieved in up to 90% patients but is highly variable and dependent on the extent of disease, type of interventions (antibiotic vs surgical debridement), and vascular supply to the affected area [8]. The treatment of osteomyelitis of the head and neck presents with its own set of complications due to aesthetic considerations and the anatomy of the area [9]. The general treatment of osteomyelitis usually involves surgical debridement of necrotic material and biopsies for the culture of affected tissue/bone (assuming the patient is an appropriate candidate) followed by appropriate antimicrobial therapy. For patients who have no residual infected bone post-debridement, a short course of antibiotics (10-14 days) directed at the identified pathogen is a reasonable approach [1]. In contrast, those patients with residual infected bone require a prolonged course of antibiotics.

In this case, the patient was initially treated with IV Unasyn (ampicillin and sulbactam) and IV fluconazole and was subsequently switched to IV cefepime, metronidazole, and fluconazole. In total, he received 13 days of IV antimicrobials and showed clinical improvement. Upon discharge, he was sent home with a novel antibiotic, omadacycline, which has been shown to be a promising candidate for osteomyelitis treatment [10]. In total, he received six weeks of antibiotic therapy with weekly complete blood count (CBC), comprehensive metabolic panel (CMP), erythrocyte sedimentation rate (ESR), and C-reactive protein (CRP) to monitor for resolution.

## Conclusions

Osteomyelitis of the maxilla is a serious infection that needs to be diagnosed and treated early in the clinical course to avoid life-threatening infections and complications such as sinus tract formation, sepsis, bone deformity, and malignancy. Similarly, it is important to identify and address pre-disposing factors to prevent future recurrences. This task can be complicated in patients who have no obvious risk factors. Additionally, further investigations into FSGS as one of the potential risk factors for pansinusitis and osteomyelitis need to be conducted.
